# A Two-Component System (XydS/R) Controls the Expression of Genes Encoding CBM6-Containing Proteins in Response to Straw in *Clostridium cellulolyticum*

**DOI:** 10.1371/journal.pone.0056063

**Published:** 2013-02-13

**Authors:** Hamza Celik, Jean-Charles Blouzard, Birgit Voigt, Dörte Becher, Valentine Trotter, Henri-Pierre Fierobe, Chantal Tardif, Sandrine Pagès, Pascale de Philip

**Affiliations:** 1 Aix-Marseille Université, CNRS, UMR7283, Marseille, France; 2 Institut für Mikrobiologie, Ernst-Moritz-Arndt Universität, Greifswald, Germany; University of South Florida College of Medicine, United States of America

## Abstract

The composition of the cellulosomes (multi enzymatic complexes involved in the degradation of plant cell wall polysaccharides) produced by *Clostridium cellulolyticum* differs according to the growth substrate. In particular, the expression of a cluster of 14 hemicellulase-encoding genes (called *xyl-doc*) seems to be induced by the presence of straw and not of cellulose. Genes encoding a putative two-component regulation system (XydS/R) were found upstream of *xyl-doc*. First evidence for the involvement of the response regulator, XydR, part of this two-component system, in the expression of *xyl-doc* genes was given by the analysis of the cellulosomes produced by a regulator overproducing strain when grown on cellulose. Nano-LC MS/MS analysis allowed the detection of the products of all *xyl-doc* genes and of the product of the gene at locus Ccel_1656 predicted to bear a carbohydrate binding domain targeting hemicellulose. RT-PCR experiments further demonstrated that the regulation occurs at the transcriptional level and that all *xyl-doc* genes are transcriptionally linked. mRNA quantification in a regulator knock-out strain and in its complemented derivative confirmed the involvement of the regulator in the expression of *xyl-doc* genes and of the gene at locus Ccel_1656 in response to straw. Electrophoretic mobility shift assays using the purified regulator further demonstrated that the regulator binds to DNA regions located upstream of the first gene of the *xyl-doc* gene cluster and upstream of the gene at locus Ccel_1656.

## Introduction

Degradation of plant cell wall polysaccharides has remarkable practical applications, especially in the renewable bioenergy sector [1], [2]. *Clostridium cellulolyticum,* an anaerobic, mesophilic and cellulolytic bacterium, isolated from decayed grass [3], is a model organism for such applications [4], [5] and for the study of plant cell wall polysaccharide degradation. This bacterium produces extracellular enzymes assembled in high-molecular-mass complexes named cellulosomes [6] that hydrolyze plant cell wall polysaccharides into simple sugars. For instance, *C. cellulolyticum* is able to grow on cellulose as sole carbon source as well as on wheat straw or soluble sugars such as cellobiose, glucose, xylose, or arabinose [7], [8]. The assembly of cellulosomes is mediated via the interaction between eight cohesin modules of a non-catalytic scaffolding protein (CipC) and the dockerin modules of eight catalytic enzymes [9], [10]. According to genome sequencing data (NC_011898.1; GI:220927459), there are 62 putative dockerin-bearing proteins that could assemble into cellulosomes [11]. Since cohesin modules were not found to be specific of a particular dockerin module, each of the catalytic subunits would randomly assemble into cellulosomes [9], [10], [12]. It has been demonstrated that the composition of cellulosomes depends on the growth substrate to be degraded [11] and on the amounts of dockerin-bearing proteins present [13].

**Figure 1:**
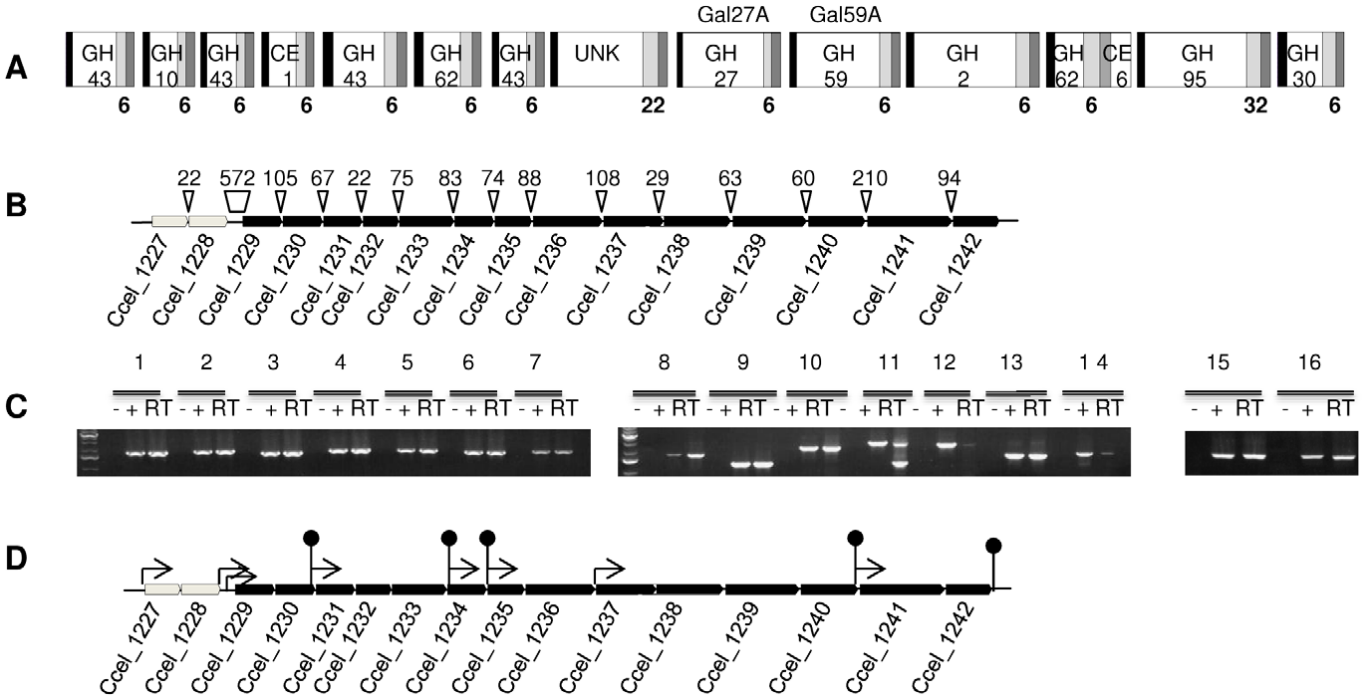
Loci Ccel_1227 to Ccel_1242 of *C. cellulolyticum* genome. A) Modular organization of *xyl-doc* genes products (loci Ccel_1229 to Ccel_1242). Signal sequence are given as black- and dockerin domains as dark grey- boxes. The catalytic domains are the white boxes in which the predicted glycoside-hydrolase (GHx) or carbohydrate esterase (CEx) family when known or UNK for a domain of unknown function are given. A bold unique number under a light grey box indicates the family of the carbohydrate binding domains found in the proteins. The family numbers are given according to CAZy database (http://www.cazy.org). B) Genetic organization of *xyd*S/R (loci Ccel_1227 and Ccel_1228 respectively, shown in light grey) and *xyl-doc* genes (from loci Ccel_1229 to Ccel_1242, shown in black). The genes are named by their locus tag. Sizes (in bp) of intergenic sequences are indicated on the black triangles. C) RT-PCR analysis of *xyd*S/R and *xyl-doc* genes. PCR amplification was performed on cDNA after RT with pairs of primers and PCR products were analyzed by electrophoresis in agarose gel (lanes RT). Primers used were localized at the end of the first gene for the direct primer and at the beginning of the second gene for the reverse primers to reveal transcriptional links between two successive genes. The pairs are as follow : 1229rtD/1230rtR (lanes 1), 1230rtD/1231rtR (lanes 2), 1231rtD/1232rtR (lanes 3), 1232rtD/1233rtR (lanes 4), 1233rtD/1234rtR (lanes 5), 1234rtD/1235rtR (lanes 6), 1235rtD/1236rtR (lanes 7), 1236rtD/1237rtR (lanes 8), 1237rtD/1238rtR (lanes 9), 1238rtD/1239rtR (lanes 10), 1239rtD/1240rtR (lanes 11), 1240rtD/1241rtR (lanes 12), 1241rtD/1242rtR (lanes 13), 1242rtD/1243rtR (lanes 14), 1227rtD/1228rtR (lanes 15), 1228rtD/1229rtR (lanes 16). −, PCRs performed on RNAs in the absence of the RT step; +, PCRs performed on genomic DNA templates. D) Genetic organization of *xyd*S/R and *xyl-doc* genes as in (A) with schematic localizations of promoters (thin arrow) and terminators (stem-loop) predicted by BPROM and FindTerm programs (http://linux1.softberry.com).

Many of the characterized cellulosomal enzymes, as well as the scaffolding protein, are encoded by a large 24-kb operon of 12 genes, called *cip-cel* operon, which is essential for cellulose degradation [14], [15]. Studies on the regulation of the expression of cellulosomal genes by the growth substrate in *C. cellulolyticum*, have been limited to the *cip-cel* operon. The expression of the *cip-cel* operon is controlled by complex mechanisms that involve carbon catabolite repression and differential mRNA maturation [15], [16]. Using mass spectrometry, most of the products encoded by the *cip-cel* operon were detected in cellulosomal preparations obtained from cultures grown on cellulose or straw-based medium [11]. A second large gene cluster of 32 kb, called *xyl-doc,* comes in addition to the *cip-cel* operon. The 14 putative cellulosomal proteins encoded by the *xyl-doc* gene cluster (loci Ccel_1229 to Ccel_1242) all display a signal sequence, a Carbohydrate-Binding Module (CBM) predicted to target hemicelluloses (either CBM6 or CBM22) and a dockerin module (Figure 1A). Furthermore, most of the catalytic domains of these enzymes, including glycoside hydrolases (GH, families 2, 10, 27, 30, 43, 59, 62, and 95) and carbohydrate esterases (CE, families 1 and 6), are predicted to be involved in hemicellulose degradation. Interestingly, these proteins are detected only in cellulosomes produced by cells grown on wheat-straw based medium [11]. Straw is a natural and complex substrate containing the different types of polysaccharides of the plant cell wall, among which cellulose, hemicelluloses and pectins. Therefore, it is relevant to hypothesize that component(s) present in straw and/or linked to its degradation induce(s) the expression of *xyl-doc* genes.

Immediately upstream of the *xyl-doc* gene cluster, genome annotation indicates the presence of genes encoding a putative two-component regulatory system (loci Ccel_1227 and Ccel_1228; Figure 1B). Two-component systems are conserved transduction mechanisms that allow organisms to respond to environmental changes [17], [18]. Our current working hypothesis is that i) the putative membrane-bound histidine kinase encoded by the gene at locus Ccel_1227 (named *xydS* for *xyl-doc* sensor) would sense the signal linked to straw and/or its degradation; ii) the putative protein encoded by the gene at locus Ccel_1228 (named *xydR* for *xyl-doc* regulator), a predicted response regulator belonging to the family of AraC/XylS-like transcriptional activators [19], would then be activated by phosphotransfer; iii) phosphorylated XydR would in turn activate the expression of *xyl-doc* genes.

In this work, we describe the involvement of the response regulator XydR (locus Ccel_1228) in the regulation of the *xyl-doc* gene cluster transcription in the presence of wheat straw. Furthermore, we show that XydR also regulates the transcription of another gene (locus Ccel_1656) encoding the only other CBM6-containing protein produced by *C. cellulolyticum*. Thus, the production of all CBM6-containing proteins in *C. cellulolyticum* is under the control of the response regulator XydR.

## Results

### Transcriptional Links between *xyl-doc* Genes Expressed in Response to Straw

As shown in Figure 1B, many of the intergenic sequences between *xyl-doc* genes are short. Taken together with the fact that many of the products of these genes were detected in the same conditions [11], we studied the transcription of these genes by RT-PCR in *C. cellulolyticum* grown in straw-based medium.

As illustrated in Figure 1C, amplification was observed between each successive gene suggesting that there is a transcriptional link from the first gene (locus Ccel_1229) to the last gene (locus Ccel_1242) of *xyl-doc* cluster. Indeed, PCR products after reverse transcription were observed (RT lanes) at the same size as the PCR products obtained using genomic DNA (positive control lanes) whereas no products were obtained when PCR reactions were performed using RNA preparations (negative control lanes). A second amplification product is observed at a lower size than expected between loci Ccel_1239 and Ccel_1240 (Figure 1C, lane 11RT); this product may be due to a secondary structure that mRNA could undergo during cDNA synthesis. In addition, weak amplifications were obtained between Ccel_1240 and Ccel_1241 (Figure 1C, lane 12RT) and between Ccel_1242 and Ccel_1243 (Figure 1C, lane 14RT) suggesting the presence of more or less weak terminators located downstream of Ccel_1240 and Ccel_1242 or processing events in these regions. It may be relevant to note that the intergenic sequence between loci Ccel_1240 and Ccel_1241 is the largest one found in the cluster. In this intergenic sequence, the use of FindTerm and BPROM programs (http://linux1.softberry.com) allowed to find a putative terminator downstream of locus Ccel_1240 and to predict a promoter with its -35 and -10 sequences downstream of this terminator and upstream of locus Ccel_1241 (Figure 1D). According to sequence annotation and results from Blouzard et al. [11] the *xyl-doc* gene cluster finishes after the gene at locus Ccel_1242. Indeed, FindTerm predicts a terminator downstream of this gene (Figure 1D). However, presence of a terminator, depending on its strength, does not exclude the possibility of a transcriptional link with the following gene, located 118-bp downstream in the same orientation.

In addition to the results on *xyl-doc* genes transcriptional links, our RT-PCR results indicate that the expression of the two genes encoding the two-component system XydS/R (at loci Ccel_1227 and Ccel_1228) is transcriptionally linked (Figure 1C, lane 15RT) and that there is also a link with the first gene of the *xyl-doc* cluster (locus Ccel_1229; Figure 1C, lane 16RT) and thus to all *xyl-doc* genes. According to the size of the intergenic sequence between loci Ccel_1228 and Ccel_1229 (572 bp, Figure 1B) and to the prediction of two putative promoters in this sequence (BPROM program), this link may be surprising (Figure 1D). However, FindTerm program failed to identify a terminator downstream of the locus Ccel_1228 (Figure 1D).

### Modification of *xyl-doc* Gene Expression by *in vivo* Overproduction of the XydR Regulator

In order to investigate the involvement of XydR in the regulation of the expression of *xyl-doc* genes, we first chose to overproduce a derivative of XydR in *C. cellulolyticum* and to analyze the effect on cellulosomes composition.

From primary sequence analysis, XydR is predicted to be a response regulator belonging to COG4753 over all its sequence. It consists of 532 amino acids with an N-terminal conserved CheY-like receiver domain of 122 amino acids belonging to the REC super family (clo09944 in Conserved Domain Database, CDD, http://www.ncbi.nlm.nih.gov/Structure/cdd/cdd.shtml) [20], and in its C terminus, a 42 amino-acid DNA binding domain belonging to helix-turn-helix motif of AraC family (clo2815 in CDD**)**. Thus, XydR, as many orthodox response regulators, has a N-terminal conserved regulatory domain, that should modulate the activity of the effector domain in response to the signal, and a DNA-binding effector domain with a helix-turn-helix motif conserved among AraC/XylS transcriptional regulators [19] at its C terminus. In this work, we chose to overproduce and to purify a truncated derivative of XydR whose activity should not be regulated by its cognate putative kinase (hypothesized to be encoded by gene at locus Ccel_1227) in response to the environmental signal. To construct this XydR derivative, the sequence encoding its N- terminal conserved regulatory domain (116 amino acids) was deleted from *xydR* gene. This deleted *xydR* gene was subsequently cloned in the expression vector pSOS954 (Table 1) [13] under the control of the thiolase gene promoter (P*_thl_*, a promoter found to be constitutive in *C. cellulolyticum*) thereby generating pSOS954Δ116 (Table 1), which was then used to transform wild-type H10 *C. cellulolyticum* strain. Two independent clones isolated from strain H10(pSOS954Δ116) (Table 1), as well as a H10(pSOSzero) strain, which contains the expression vector with no expression cassette (Table 1), and H10 strain were grown in cellulose- or straw-based medium for cellulosomes preparation.

**Table 1:** Bacterial strains and plasmids used in this study.

Strain or plasmid	Relevant feature(s)	Source or reference
*E. coli* BL21(DE3)	F^−^ *omp*T *dcm* hsdS_B_(r_B_^−^ m_B_^−^) *gal lon* λDE3	Novagen
*E. coli* DH5α	F^−^Φ80 *lac*ZΔM15Δ(*lac*ZYA-*arg*F) U169 *rec*A1 *end*A1 *hsd*R17(r_K_^−^ m_K_^+^) *pho*A *sup*E44 *thi*-1 *gyr*A96 *rel*A1 λ^−^	Invitrogen
*E. coli* JM109	*rec*A1 *mcr*B^+^ *hsd*R17	Promega
*C. cellulolyticum* H10	wild-type strain, ATCC35319/DSM 5812	DSMZ
*C. cellulolyticum* MTL1228	ATCC35319 derivative, *xyd*R ::intron, Em^r^	This study
pAM120	*tet*M from *E. faecalis* carrying vector; Tc^r^	[46]
pET22b(+)	*E. coli* expression vector (T7 promoter); Ap^r^	Novagen
pET1230GH10	pET22b(+) derivative carrying 0.9-kb NdeI-XhoI fragment of locus Ccel_1230 encoding GH10 domain	This study
pET1234	pET22b(+) derivative carrying the 1.5-kb NcoI-XhoI fragment of locus Ccel_1234	This study
pET1237	pET22b(+) derivative carrying the 1.7-kb NdeI-XhoI fragment of locus Ccel_1237	This study
pET1656Unk	pET22b(+) derivative carrying the 2.2-kb NdeI-XhoI fragment of locus Ccel_1656 encoding unknown domain	This study
pGEMT-Easy	*E. coli* cloning vector; Ap^r^	Invitrogen
pJIR418	*cat*P from *C. perfringens* carrying vector, Tm^r^	[47]
pMAL-c2x	*E. coli* MBP fusion vector (*tac* promoter, *mal*E); Ap^r^	New England Biolabs
pMALΔ116	pMAL-c2x derivative carrying 1.2-kb BamH1-Pst1 fragment containing deleted *xydR*	This study
pMTL007	*E. coli/Clostridium* shuttle vector (ColE1, pCB102) Ll.*ltr*Bintron (*erm*BtdRAM2) under the controlof P*_fac_*, *ltr*A; Cm^r^/Tm^r^	[21]
pMTL007xydR	pMTL007 derivative targeting *xyd*R (locus Ccel_1228)	This study
pSOS954	*E. coli/Clostridium* shuttle expression vector, (ColE1, pIM13), P*_thl_*; expression cassette from*C. acetobutylicum*, Ap^r^, Em^r^	[13]
pSOSzero	*E. coli/Clostridium* shuttle vector (ColE1, pIM13); Ap^r^, Em^r^	[13]
pSOSzeroTm	pSOSzero derivative*;* Ap^r^, Tm^r^	This study
pSOSzeroTc	pSOSzeroTc derivative*;* Ap^r^, Tc^r^	This study
pSOS954Δ116	pSOS954 derivative carrying 1.3-kb BamH1-NarI fragment containing P*_thl-_xydR*; Ap^r^, Em^r^	This study
pSOS955	pSOS954 derivative, *P_thl_*; expression cassette from *C. cellulolyticum;* Ap^r^, Tc^r^	Generous gift from S. Perret
pSOS955Δ116	p955 derivative carrying 1.3-kb BamH1-NarI fragment containing P*_thl-_xydR;* Ap^r^, Tc^r^	This study

Ap^r^, ampicilline resistance; Tc^r^, tetracycline resistance, Cm/Tm^r^, chloramphenicol/thiamphenicol resistance; Em^r^, erythromycine resistance.

**Figure 2:**
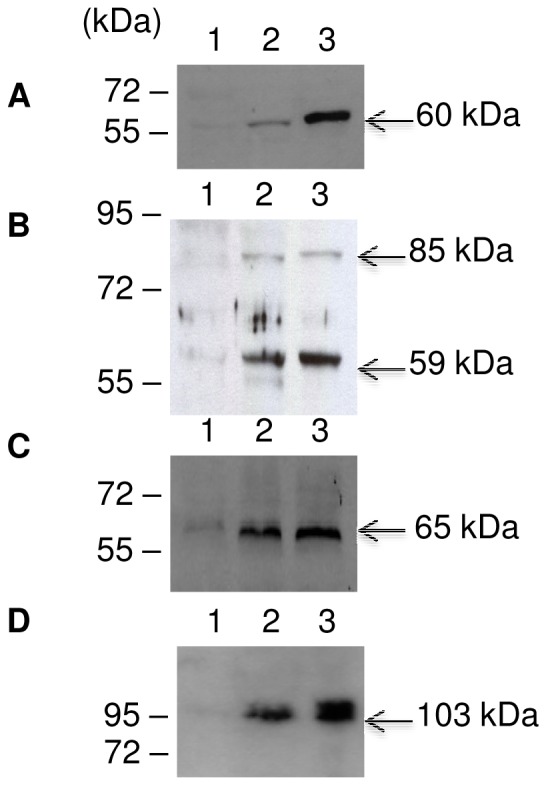
Detection of CBM6-containing proteins by western blot in cellulosomes. 25 µg of cellulosomes produced by the control strain H10(pSOSzero) grown in cellulose-based medium (lanes 1) or straw-based medium (lanes 2) and the overproducing truncated regulator strain H10(pSOSΔ116) grown on cellulose-based medium (lanes 3) were subjected to SDS-PAGE and transferred to nylon membranes. Western blots were probed with antibodies directed against (A) GH10 domain of the product of the gene at locus Ccel_1230, (B) product of gene at locus Ccel_1234, (C) Gal27A (product of gene *gal27A*, locus Ccel_1237), and (D) unknown domain of the product of the gene at locus Ccel_1656. Arrows indicate the expected molecular mass of the corresponding mature proteins.

The secreted cellulosomal proteins (harboring dockerin or cohesin modules) obtained in each culture conditions, were firstly separated using SDS-PAGE and secondly identified with liquid chromatography coupled to tandem mass spectrometry (LC MS/MS). Comparison of the compositions of the cellulosomes obtained from H10 strain grown on straw-based medium and from H10(pSOSzero) strain, grown on cellulose-based medium confirmed our previous results [11] (data not shown). Among all the carbohydrate active enzymes (CAZymes), few were detected in higher amounts in cellulosomes produced by H10 strain grown on straw-based medium compared to cellulosomes produced by H10(pSOSzero) strain grown on cellulose-based medium; these are listed in Table 2. In addition to the products of *xyl-doc* cluster (loci Ccel_1229 to Ccel_1242), the product of the gene at locus Ccel_1656 was produced in higher quantities in the straw-based medium compared to the cellulose-based medium. This last product is a secreted dockerin-containing protein of unknown function harboring a CBM6, which is predicted to target hemicelluloses as the carbohydrate-binding modules present in the products encoded by *xyl-doc*. Interestingly, in cellulosomes produced by H10(pSOS954Δ116), overproducing the truncated regulator, grown on cellulose-based medium, products of *xyl-doc* genes (except the products of the genes at loci Ccel_1231 and Ccel_1232) and the CBM6-containing protein, encoded by the gene at locus Ccel_1656, are detected in higher quantities compared to H10(pSOSzero) grown on cellulose (Table 2). In cellulosomes produced by these strains grown on cellulose, the quantity of the scaffolding protein CipC (gene at locus Ccel_0728) is approximately the same (2,131 and 2,163 spectral counts for clone 1 and 2 versus 1,952 for control strain). Thus, the high amounts of proteins detected reflect a lack of regulation when the truncated regulator is overproduced. Our results strongly support the hypothesis that XydR is involved in the regulation of the production of the CBM6-containing proteins synthesized by *C. cellulolyticum* when growing on straw. To confirm this indication, we performed western blot analyses and thus raised antibodies directed against products of genes at loci Ccel_1230 (60 kDa), Ccel_1237 (65 kDa), Ccel_1234 (59 KDa), and Ccel_1656 (103 kDa). As seen in Figure 2, each antibody targeted a protein of its predicted molecular mass. The antibody directed against the product of the gene at locus Ccel_1234, a GH62-containing protein, however also probed a protein which could be the product of the gene at locus Ccel_1240 (85 kDa, Figure 2C), since this protein is also a GH62-containing protein. All the target proteins were detected in cellulosomes produced by H10(pSOSzero) grown on cellulose but at barely detectable levels, whereas these proteins seemed much more abundant in cellulosomes produced when the strain was grown on straw-based medium or when the regulator overproducing strain H10(pSOSΔ116) was grown in cellulose-based media (Figure 2).

**Table 2:** Detection of the products of *xyl-doc* and CBM6-containing proteins by LC-MS/MS in cellulosomes^a^.

Locus tag	Predicted function^b^	Mass^c^ (kDa)	Quantitative value^d^			
			straw	cellulose	cellulose	cellulose
			H10	H10 (pSOSzero)	H10 (pSOSΔ116) clone 1	H10 (pSOSΔ116) clone 2
Ccel_0728	CipC Scaffolding protein	156	831	1952	2131	2163
Ccel_1229	xylanase/arabinosidase	58	63	15	64	126
Ccel_1230	xylanase	60	90	35	152	158
Ccel_1231	xylanase	57	18	2	1	1
Ccel_1232	feruloyl esterase	53	58	ni	1	0
Ccel_1233	xylosidase/arabinofuranosidase	82	42	14	98	119
Ccel_1234	α-arabinofuranosidase	59	80	47	103	162
Ccel_1235	xylanase	56	46	ni	4	8
Ccel_1236	unknown	105	8	4	23	51
Ccel_1237	Gal27A α-galactosidase	65	20	9	52	70
Ccel_1238	Gal59A α-galactosidase	122	10	ni	16	20
Ccel_1239	β-galactosidase	110	18	12	55	37
Ccel_1240	arabinofuranosidase/acetyl-xylan-esterase	85	89	48	150	192
Ccel_1241	α-fucosidase	127	8	2	47	12
Ccel_1242	xylanase/glucuronoxylanase	68	30	15	80	61
Ccel_1656	unknown	103	35	2	134	119

a Cellulosomes were produced by H10, H10(pSOSzero), H10(pSOS954Δ116) grown in cellulose- or straw-based media.

b As given in Blouzard et al. 2010 [11].

c Theoretical masses.

d Quantitative values given as the spectral counts found by LC MS/MS analysis for identified proteins. ni, not identified.

### Regulation of CBM6-containing Protein Synthesis Occurs at the Transcriptional Level

**Figure 3:**
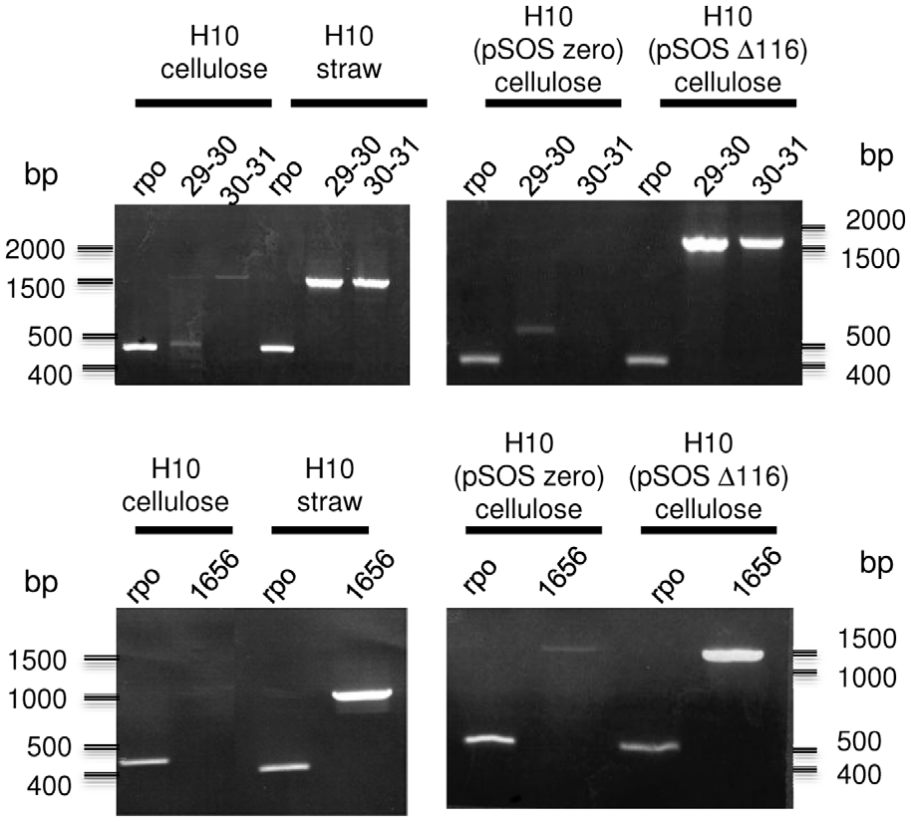
RT-PCR amplification of mRNA produced by *C. cellulolyticum* wild type or overproducing constitutive regulator. mRNA were prepared from cultures of H10, H10(pSOSzero), and H10(pSOS954Δ116) grown on cellulose or straw as indicated and reverse transcribed. PCR was performed on the cDNA with the following pairs of primers (Table S1): RPO-F/RPO-R (Table S1) targeting a housekeeping gene used for standardization and quantification of the induction (RPO), 1229rtD/1229rtR (Table S1) targeting end of the first gene and beginning of the second gene of *xyl-doc* cluster (29–30), 1230rtD/1231rtR (Table S1) targeting end of the second gene and beginning of the third gene of *xyl-doc* cluster (30–31), and 1656rtD/1656rtR (Table S1) targeting upstream sequence and beginning of the gene at locus Ccel_1656 (1656).

XydR regulator is predicted to contain a helix-turn-helix domain typical of AraC/XylS transcriptional activators. Thus, induction of the expression of *xyl-doc* genes and of the unknown CBM6-containing protein-encoding gene were studied by RT-PCR with mRNAs prepared from H10 strain grown in cellulose- and straw-based media, and mRNA prepared from H10(pSOSΔ116) strain grown in cellulose-based medium. In all experiments, the level of expression of the gene *rpoD*-like (locus Ccel_0541), encoding Sigma70 RNA-polymerase subunit, was used for standardization of the amplification results to obtain semi-quantitative data. The study of the expression of *xyl-doc* gene cluster, limited to the first 3 genes of the cluster (loci Ccel_1229 to Ccel_1231), indicated clearly an over expression of *xyl-doc* genes upon use of straw as a growth substrate in wild-type strain and upon use of cellulose as a growth substrate in Δ116 overproducing strain (Figure 3 top). Same patterns of expression were obtained for the gene at locus Ccel_1656 (Figure 3 bottom). Thus, regulation, induced by the presence of straw, occurs at the transcriptional level and seems to involve XydR as a transcriptional regulator.

### Effect of the Inactivation of the Regulator-encoding Gene *xydR* on the Expression of *xyl-doc* Genes and Gene at Locus Ccel_1656

The interruption of the target gene *xydR* (locus Ccel_1228*)* encoding the response regulator XydR was performed using ClosTron technology [21]. *C. cellulolyticum* clones interrupted in *xydR* were named MTL1228. Molecular analysis by PCR and Southern blot hybridization confirmed the integration of the intron within *xydR* and the curation of replicative pMTL007xydR (data not shown). The mutant strain MTL1228 was complemented *in trans* with pSOS955Δ116, allowing expression, from the constitutive thiolase gene promoter, of the sequence encoding the truncated regulator (Table 1). In order to exclude any plasmid effect on the phenotype observed, a strain harboring pSOSzeroTc (Table 1), that does not express *xydR* derivative, was also constructed.

**Figure 4:**
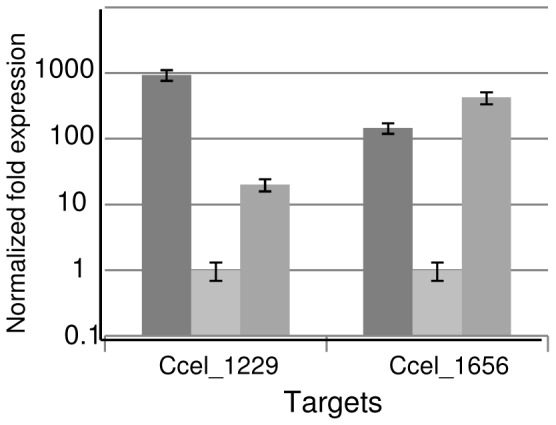
qRT-PCR analysis of mRNA produced by *xyd*R knock-out strain. mRNA were prepared from cultures in straw-based medium of wild-type (H10), MTL1228(pSOSzeroTc) and MTL1228(pSOS955Δ116) strains and reverse transcribed. qPCR was performed on the cDNA with the following pairs of primers :1229qRT-F/1229qRT-R (Table S1) targeting the first gene of *xyl-doc* cluster at locus Ccel_1229, 1656qRT-F/1656qRT-R (Table S1) targeting the gene at locus Ccel_1656. Levels of expression of genes at loci Ccel_1229 and Ccel_1656 are given after standardization with the level of expression of *rpo*D (using the primers RPO-F and RPO-R, Table S1). Error bars indicate the standard deviation of three independent qPCR reactions. Dark grey represents the results obtained with mRNA from H10 strain, light grey are the ones with mRNA from MTL1228(pSOSzeroTc) strain and grey ones with mRNA from MTL1228(pSOSΔ116) strain.

Both strains, MTL1228(pSOSzeroTc) and MTL1228(pSOS955Δ116), together with the wild-type H10 strain, were grown on straw-based medium and total RNAs were extracted and submitted to qRT-PCR to study the expression of the first gene of *xyl-doc* cluster (locus Ccel_1229) and of the gene at locus Ccel_1656 (Figure 4). In *xydR* mutant strain, MTL1228(pSOSzeroTc), expression of these two genes is greatly diminished, whereas in the complemented strain, MTL1228(pSOS955Δ116), expression level of the first gene of the *xyl-doc* gene cluster, was approximately 11-fold higher and the expression level of the gene at locus Ccel_1656 was recovered back to wild-type level.

The results show unambiguously the involvement of XydR in the expression of the genes at loci Ccel_1229 and Ccel_1656. As transcriptional links were shown between gene at locus Ccel_1229 and the other *xyl-doc* genes, it can be concluded that the regulator XydR is involved in the expression of *xyl-doc* genes and of the gene at locus Ccel_1656.

### Binding of the Regulator XydR Upstream of its Target Genes

**Figure 5:**
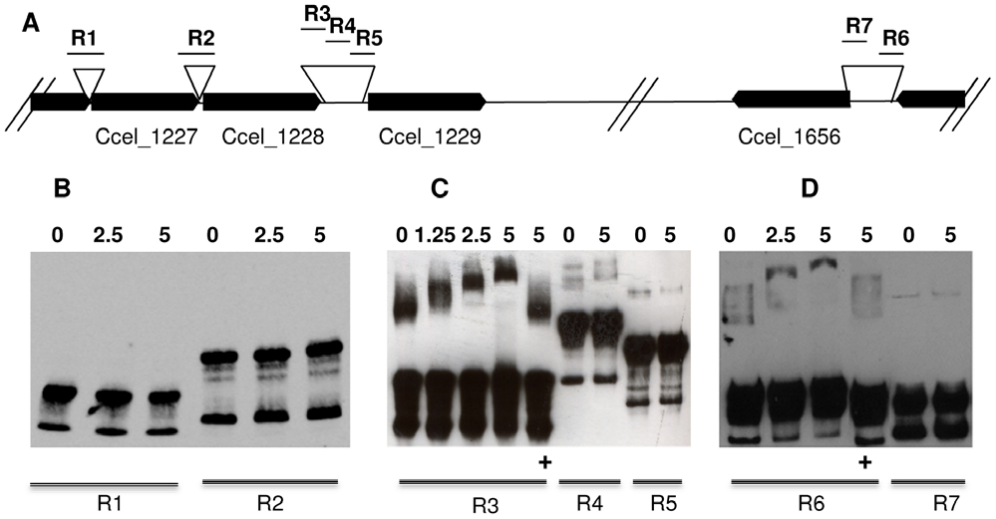
Binding of a truncated XydR regulator to target regions. A) Schematic representation of targeted regions [R1 (309 bp), R2 (347 bp), R3 (272 bp), R4 (429 bp), R5 (345 bp), R6 (246 bp), and R7 (240 bp)] upstream genes at loci Ccel_1227, CCel_1228, Ccel_1229, and Ccel_1656. The loci of the different genes are represented as black arrows. B, C and D) Electromobility shift assays were carried out with 20 fmol of 3′ OH biotin-labeled regions (R1, R2, R3, R4, R5, R6, and R7) without any protein or with various amounts of purified MBP-Δ116 which are indicated in nM on top of the lanes. Competitive inhibition of the bindings were performed with 5 nM of MBP-Δ116, 20 fmol of 3′ OH biotin-labeled of regions R3 and R6 and 2 pmol of unlabeled R3 and R6 regions respectively (lanes with+at the bottom).

In order to clearly identify the binding regions of the regulator XydR, we chose to perform Electro-Mobility Shift Assays (EMSA) using the truncated XydR derivative. Attempts to produce and purify this derivative using His- or Strep-tags did not succeed. The different proteins were found to be produced in *E. coli* mainly in an insoluble form. However, maltose binding protein (MBP) fused to the N terminus of truncated XydR derivative successfully yielded a highly soluble protein (MBP-Δ116). Different DNA sequences upstream of i) the sensor- encoding gene (locus Ccel_1227); ii) the regulator- encoding gene (locus Ccel_1228); iii) the first gene of *xyl-doc* gene cluster (locus Ccel_1229); and iv) the gene encoding the CBM6-containing protein of unknown function (locus Ccel_1656) were assayed in EMSAs (Figure 5). The long non-coding sequences present upstream of genes at loci Ccel_1229 and Ccel_1656 were shortened as described in Material and Methods and shown in Figure 5A. As observed in Figure 5B, no binding of MBP-Δ116 was observed upstream of the two genes encoding the two-component system (loci Ccel_1227 and Ccel_1228), whereas binding is observed for part of the regions upstream of the first gene of *xyl-doc* gene cluster (R3, upstream of locus Ccel_1229, Figure 5C) and upstream of the gene encoding the CBM6-containing protein of unknown function (R6, upstream of locus Ccel_1656, Figure 5D). Indeed, for these regions, migration is hindered proportionally to the amount of MBP-Δ116 incubated with the labeled DNA. These bindings were found to be specific as shown by the competition assays done with large amounts of the corresponding unlabeled DNA (Figure 5C and 5D, lanes labeled +). Thus, our mobility shift assays unambiguously show that the regulator XydR binds *in vitro* to DNA regions upstream of the genes whose expression was shown to be regulated *in vivo* (loci Ccel_1229 and Ccel_1656). As the gene at locus Ccel_1229 is the first gene of a cluster of genes transcriptionally linked, the expression of all the genes of this cluster should be controlled by XydR.

## Discussion

Previous studies have shown that C. *cellulolyticum* could adapt the composition of the cellulosomes it produces depending on the growth substrate [11]. In particular, products of *xyl-doc* gene cluster are synthesized when the strain grows on straw. This cluster of genes is predicted to encode all but one CBM6-containing proteins of *C. cellulolyticum*. In the present work, we demonstrate the involvement of a regulator XydR, whose gene is located directly upstream of *xyl-doc* cluster, in the regulation of the expression of this gene cluster and of the gene encoding the last CBM6-containing protein (locus Ccel_1656).

From primary sequence analysis, XydR is predicted to be the response regulator of a prototypical regulatory system [17], [18], the second component of the system being encoded by the gene directly upstream of *xydR* encoding a histidine kinase sensor (*xydS* at locus Ccel_1227). Moreover, XydR is predicted to belong to the family of AraC/XylS transcriptional activators because the helix-turn-helix motif of AraC is present at its C terminus extremity. Depending on the mechanism of activation and the consequences of phosphorylation of the regulator domain, a truncated regulator depleted from its regulator domain can act as a constitutively active effector. For instance, overproduction of truncated FixJ or DevR proteins can induce target-gene expression independently of the environmental activation signal [22], [23] and truncated derivatives of DevR, FixJ, Spo0A, FruA, XylS were shown to bind to their target sequences upstream the genes which they regulate [23], [24], [25], [26], [27], [28]. In addition, it has also been described that derivatives of AraC or RhaS, depleted of their sugar binding domain, activate transcription of their target genes [29], [30]. As for many of these regulators, the regulator domain of XydR seems to act negatively on its effector domain. Indeed, overproduction of truncated XydR (depleted of its regulator domain) allowed production of all targeted proteins (CBM6-containing proteins) independently of the presence of the inducer (straw) in the growth-medium. Moreover, the truncated protein consisting only of the effector domain, was shown to bind specifically to target DNA sequences located upstream of regulated genes. An additional common feature of transcriptional activators belonging to AraC/XylS-family is their difficulty to be overproduced in a soluble form [31] that triggered the use of a MBP-fusion protein like for instance PerA, XylS, or PchR [32], [33], [34].

As shown by qRT-PCR analyses of mRNAs from wild-type strain (H10) and from *xydR*-disrupted strain (MTL1228) complemented or not by *xydR in trans*, XydR is involved in the regulation of the expression of genes at loci Ccel_1229 and Ccel_1656. Only partial recovery of the expression of the gene at loci Ccel_1229, however, was observed in MTL1228 strain complemented by *xydR in trans*. We hypothesize that this partial complementation could be due to the disruption of *xydR* (at locus Ccel_1228) in strain MTL1228 leading to i) a polar effect as expression of genes at loci Ccel_1228 and Ccel_1229 is linked, or ii) a modification of the DNA conformation of the binding site of XydR.

Electromobility shift assays results have shown that the effector domain of XydR binds to DNA regions upstream to loci Ccel_1229 and Ccel_1656. The binding occurs between minus 327 bp and minus 599 bp, and between minus 237 bp and minus 483 bp from the start codons of the genes at loci Ccel_1229 and Ccel_1656, respectively. In these regions, promoters were predicted by BPROM program with clear −35 consensus and −10 consensus sequences located at minus 415 bp (for −10 consensus) and at minus 299 bp (for −10 consensus) from the start codons of these genes, respectively. Sequence comparison of these two regions highlighted many common features such as inverted repeats, direct repeats, and sequence homologies upstream and on the predicted −35 boxes (Table 3). Numerous transcriptional activators belonging to AraC/XylS family have been studied, however, no consensus DNA binding site could be identified [19], [35]. Recently, Schuller et al. [36] published sequence logos for AraC/XylS transcriptional activators depending on the biological process in which they are involved. However, among the common sequences presented in Table 3, no sequence resembles to the DNA logo predicted for transcriptional regulators likely involved in plant cell wall degradation [36].

**Table 3:** Common DNA sequences upstream of loci Ccel_1229 and Ccel_1656.

Loci	Sequences^a^	Location^b^
Ccel_1229	TAACAA(N_15_)TACAA	−118
Ccel_1656	TAACAA(N_13_)TACAA	−140
Ccel_1229	ATAAA(N_10_)ATTTT	−85
Ccel_1656	ATAAA(N_8_)ATTTT	−114
Ccel_1229	CAAAA(N_14_)CAAAA	−39
Ccel_1656	CAAAA(N_15_)CAAAA	−39
Ccel_1229	AAAAT(N_2_)GATA	−72
Ccel_1656	AAAAT(N_0_)GATA	−69
Ccel_1229	AAAAT(N_7_)AAAAT	−56
Ccel_1656	AAAAT(N_10_)AAAAT	−58
Ccel_1229	GATTTATA(N_6_)TGGAAA(N_3_)TTTACA	−9
Ccel_1656	GATTTATA(N_6_)TGGAAA(N_3_)TTTACA	−9

a given from 5′ extremity to 3′extremity.

b distance in base pair from predicted −10 consensus sequence (BPROM;http://linux1.softberry.com).

The gene at locus Ccel_1229, whose expression is controlled by XydR, is the first gene of the cluster of 14 genes (called *xyl-doc*) from locus Ccel_1229 to locus Ccel_1242. Results of RT-PCR analyses indicate a transcriptional linkage between successive genes in the cluster. Such linkage is not surprising considering the short intergenic regions between most of these genes and is consistent with the complementary predicted functions of *xyl-doc* gene products, which seem to be all involved in hemicelluloses degradation [11]. Differential production of the corresponding proteins has, however, been observed. BPROM and FindTerm programs allowed identification of putative internal terminators (downstream of loci Ccel_1230, Ccel_1233, Ccel_1234, Ccel_1240 and Ccel_1242; Figure 1D) and putative promoters (upstream of loci Ccel_1231, Ccel_1234, Ccel_1235, and Ccel_1241; Figure 1D). In addition, our results indicate a transcriptional linkage between this cluster and the genes encoding the two-component system XydS/R located directly upstream and involved in the regulation of *xyl-doc* expression. Such linkage is a common feature among genes encoding two-component regulation systems and their targets [17]. Another common feature is the regulation, by the two-component system, of the expression of its own genes. Our RT-PCR results indicate, however, that expression of *xydRS* is not regulated by XydR (data not shown), and we failed to detect a target binding site upstream of *xydS* gene. We thus hypothesize that i) at least genes from locus Ccel_1227 to locus Ccel_1242 are transcribed constitutively, at a low basal level on a very long transcript; ii) in the presence of straw, XydR/S allows transcription of *xyl-doc* genes (loci Ccel_1229 to Ccel_1242); iii) other promoters or regulatory events such as mRNA processing, and/or RNA/protein differential stabilities, fine tune the regulation of the expression of the numerous genes of the *xyl-doc* cluster. Existence of a long transcript and regulation by processing events have already been described in *C. cellulolyticum* concerning the expression of *cip-cel* operon which encodes the major cellulosomal components essential for cellulose degradation [14], [15].

Whether or not this “*xyl-doc*” very long transcript starts at Ccel_1227 and finishes at Ccel_1242 is not well established. Indeed, no terminator has been found upstream of the gene at locus Ccel_1227 and RT-PCR results do not clearly establish that there is no linkage between the gene at locus Ccel_1242 and the downstream gene at locus Ccel_1243 which encodes a small hypothetical protein. In addition, the genes located directly upstream of *xydSR* are predicted to encode components of ABC-transporter like transporters and permeases and among the genes that are further on downstream of *xyl-doc*, some encode putative other enzymes involved in plant-cell wall degradation and putative components of ABC-transporters. It may be worth noticing that the overall organization of *xyl-doc* gene cluster and its surroundings is found in two of the other *Clostridium* whose genome sequence data are available: *C. papyrosolvens* DSM 2782 (NZ_ACXX02000000; GI:326205062) and *Clostridium* sp BNL1100 (NC_016791; GI:376259380) but neither in *C. cellulovorans* 743B [37] (NC_014393; GI:302872922) nor in *C. thermocellum* ATCC2405 or DSM1313 (NC_009012; GI:125972525 or NC_017304; GI:385777386 respectively).

Another interesting feature concerning the genomic organization is that in *C. cellullolyticum,* among the 12 pairs of genes predicted to encode a two-component system with an AraC-like regulator, one is surrounded by genes encoding putative components of ABC-transporter/permease and the other 11 are surrounded by genes predicted to encode both components of ABC-transporter/permease and carbohydrate active enzymes. One of these systems (encoded by genes at loci Ccel_0148 and Ccel_0147) present identities with YesM/N of *Paenibacillus* sp JDR-2 (30% and 32%, respectively) which is described as the system regulating aldouronate utilization, resulting from depolymerization of methylglucuronoxylan, a hemicellulose [38]. Co regulation of ABC transporter- and hydrolase-encoding genes, which has been described recently in actinobacteria for the regulation of cellulose degradation and entry of cellobiose [39], could be a general scheme of regulation of the degradation of polymeric growth substrates and entry of resulting product(s).

In addition to the *xyl-doc* gene cluster, XydR regulates the expression of an isolated gene, at locus Ccel_1656, which encodes a secreted 951-amino acid protein harboring in its C terminus a family 6 carbohydrate binding module (CBM6) predicted to bind β-1,4-xylan, and a type 1-dockerin module allowing its interaction with the scaffolding protein CipC. The role of this protein is unknown; indeed most of its primary sequence (721 amino acids) does not share any significant identity with proteins of known function(s). With the products of the *xyl-doc* gene cluster, however, it may be required for efficient hemicellulose degradation. Indeed, low expression level of *xyl-doc* gene cluster and of the gene at locus Ccel_1656, as observed in *xydR* mutant strain (MTL1228) severely impaired its growth in a straw-based medium (data not shown).

As a model, we propose that the sensor XydS is involved in sensing the signal present in straw and/or linked to its degradation. This signal should be generated by the activity of products encoded by *xyl-doc* genes and/or gene at locus Ccel_1656 transcribed at basal level. In response to this signal, XydR would be activated by phosphotransfer and in turn would activate transcription of *xyl-doc* genes and gene at locus Ccel_1656 to efficiently degrade hemicelluloses. In addition, we hypothesize that expression of specific ABC transporter-encoding genes (located upstream and downstream of *xyl-doc* cluster) could also be regulated by XydR for efficient uptake of the soluble sugars resulting from hemicellulose degradation.

## Materials and Methods

### Bacterial Strains, Plasmids, Media, and Growth Conditions

The bacterial strains and plasmids employed in this study are listed in Table 1. *Escherichia coli* strains were grown in either Luria-broth or Luria-agar in the presence of ampicillin (100 µg/mL) or chloramphenicol (34 µg/mL). *C. cellulolyticum* H10 was grown anaerobically at 32°C in basal rich medium for strain constructions [40] or minimal medium for regulation studies [16], supplemented with cellobiose (2 g/L; Sigma-Aldrich), cellulose (5 g/L; Sigmacell cellulose type 20 from Sigma-Aldrich), or hatched wheat straw (5 g/L; Valagro). Electrotransformation of *C. cellulolyticum* was performed as described previously [13], [41], [42] and recombinant clones were selected on solid basal medium supplemented with agar (15 g/L), cellobiose (2 g/L) and with appropriate antibiotic(s) (erythromycin 2.5 or 5 µg/L, thiamphenicol 30 µg/mL, and/or tetracycline 5 µg/mL) under anaerobic atmosphere of a glove box (N_2_/H_2_, 95/5 [vol/vol]) after incubation in anaerobic jars under 2×10^5^ Pa of an N_2_/CO_2_ (80/20 [vol/vol]) atmosphere.

The vectors used in *E. coli* were pGEMT-Easy for cloning a PCR fragment in DH5α and pET22b(+) for production and purification of proteins appended with His tags in BL21(DE3) and pMAL-c2x to produce and purify fusion proteins with maltose binding protein (MBP) in JM109. Shuttle expression vector pSOS954 and its derivative pSOS955 were used to express genes in *C. cellulolyticum*. pSOSzero and its derivatives were used for the construction of control strains. Lastly, a pMTL007 derivative was used to perform targeted mutagenesis of *C. cellulolyticum* (H10) to give mutant strain MTL1228.

### Production and Purification of Proteins in *E. coli*

The DNA encoding a truncated form of XydR regulator was amplified from genomic DNA of *C. cellulolyticum* by PCR using primers MBP-Δ116F and MBP-Δ116R (Table S1). These primers contained BamHI and PstI sites allowing cloning, in BamHI and PstI digested pMAL-c2x. The resulting plasmid pMALΔ116, allowed the production of Δ116 fused at its N-terminus to maltose binding protein (MBP-Δ116). This protein was produced and purified as follows: *E. coli* JM109 (pMALΔ116) was grown at 37°C with shaking to an optical density at 600 nm of 2. Isopropyl-B-D-thiogalactopyranoside (IPTG) was added to a final concentration of 100 µM and the culture was incubated with shaking at 25°C for 15 hours. The cells were then harvested by centrifugation (8,000 rpm for 10 min), resuspended in binding buffer (20 mM Tris pH 7.4, 200 mM NaCl, 1 mM EDTA) and broken using a French press. After centrifugation, MBP-Δ116, present in the supernatant, was purified using an amylose column (New England Biolabs) as described by the manufacturer. The fusion protein was then dialyzed against distilled water and concentrated by ultrafiltration in an Amicon cell (membrane cutoff 10 kDa). Estimation of its concentration was done by measuring absorbance at 280 nm. Purified MBP-Δ116 was used for electromobility shift assays after storage at −80°C.

For the production of antibodies directed against products of genes at loci Ccel_1234 and Ccel_1237, entire mature proteins were produced, whereas for the production of antibodies directed against products of genes at loci Ccel_1230 and Ccel_1656 only the catalytic domains (respectively GH10 and unknown domains) were produced. Primer pairs 1234-NcoF/1234-XhoR, 1237-NdeF/1237-XhoR, 1230GH10-NdeF/1230GH10-XhoR, and 1656UNK-NdeF/1656UNK-XhoR (Table S1) were used to amplify and add corresponding restriction sites for cloning in pET22b(+) to give the pET derivatives listed in Table 1. After transformation of *E. coli* BL21 (DE3), the overproduction of these proteins and the production of cellular extracts were performed as previously described for the production of MBP-Δ116. However, the purification step was performed using a nickel column (Ni-CAM™ HC RESIN; Sigma) as described by the manufacturer. The purified proteins were sent to Proteogenix (products of genes at loci Ccel_1234 and Ccel_1237) or Eurogentech (derivatives of products of the genes at loci Ccel_1230 and Ccel_1656) for polyclonal antibody production.

### *C. cellulolyticum* Mutant Strain Construction

The interruption of the target gene *xyd*R (locus Ccel_1228*)* encoding the response regulator XydR was performed using ClosTron technology [21]. Target site in *xydR* and primers IBS-xydR, EBS1d-xydR and EBS2-xydR used for retargeting Ll.*Ltr*B intron carried by pMTL007 (Table S1 and Table 1) were designed in line with computer algorithm available at the Sigma-Aldrich website (www.sigmaaldrich.com/TargeTron). A 353-bp PCR product containing the modified IBS, EBS1d and EBS2 sequences was amplified and assembled by overlapping PCR using IBS-xydR, EBSS1d-xydR, EBS2-xydR and EBS universal and then cloned into the HindIII and BsrGI sites of pMTL007, generating pMTL007xydR (Table 1).

Wild-type strain of *C. cellulolyticum* (H10) was electrotransformed, as previously described, with pMTL007xydR treated with MspI methylase [41], [42] and thiamphenicol resistant clones carrying replicative pMTL007xydD were first selected. In a second step, the integration event was selected in erythromycin-containing basal medium after induction by 3 mM IPTG. *C. cellulolyticum* strain interrupted in *xyd*R was named MTL1228 (Table 1).

### Production of Truncated Regulator in *C. cellulolyticum*

The DNA encoding the truncated form of XydR regulator (Δ116) was amplified from genomic DNA of *C. cellulolyticum* by PCR using primers pair 1228Δ116UpBam/1228DownNar (Table S1). These primers introduced BamHI and NarI sites allowing cloning, after digestion, in BamHI and NarI digested pSOS954 (Table 1). The resulting pSOS954Δ116, constructed in *E. coli* DH5α strain, was then introduced into *C. cellulolyticum* H10 by electrotransformation after methylation as previously described. Erythromycin resistant clones H10(pSOS954Δ116) were selected to study the effect of a constitutive overproduction of truncated XydR.

For the complementation experiment of MTL1228 mutated strain, the same vectors as in the previous experiments could not be used as they carry the same selection marker than the mutation present in the genome. Thus, equivalent constructs were performed in a tetracycline resistant derivative of pSOS954 (pSOS955Δ116, Table 1). The cloning vector (pSOS955) was a generous gift of S. Perret in which *tet*M gene from Tn*916* of *Enterococcus faecalis* (AAB01101) has been cloned in place of *mls*R of *Bacillus subtilis* (AY187686). As a control for the complementation experiment, a tetracycline resistant pSOSzero derivative, with no expression cassette was constructed (pSOSzeroTc, Table 1) in a two-step manner. First, primers catdir and catrev (Table S1) were used to amplify *catP* from *C. perfringens* carried by pJIR418 (Table 1). The fragment was then digested by SacI and ClaI and cloned at corresponding sites in pSOSzero to give pSOSzeroTm (Table 1). Secondly, primers tetd and tetr (Table S1) were used to amplify *tet*M from pAM120 (Table 1). The fragment was then digested by SmaI and ClaI and cloned at EcoRV and ClaI sites of pSOSzeroTm to give pSOSzeroTc (Table 1).

### RNA Isolation and Reverse Transcriptase Polymerase Chain Reaction (RT-PCR)

Total RNA was isolated using High Pure RNA Isolation kit (Roche Applied Science) from *C. cellulolyticum* wild type and mutant strains that were grown until mid- to late- exponential phase in either cellulose- or straw-based minimal medium. DNase I treatment (Ambion, Life Technologies) was used to remove the contaminating genomic DNA. cDNA synthesis was performed using SuperScript III reverse transcriptase and random primers as recommended (Invitrogen, Life Technologies) from 250 ng of total RNA. Finally, PCR was performed on 2.5 ng of cDNA using EmeraldAmp Max PCR master mix (Takara) and specific primer pairs depending on the gene studied (Table S1 and legend to Figure 1C).

### qRT-PCR

Quantification of cDNAs was carried out with the SsoFast Evagreen Supermix 2X kit (BioRad), according to the manufacturer’s protocol. Complementary DNA was mixed with 0.5 µM of each primer in 15 µl final volume. Pairs of primers used (1229qRT-F/1229qRT-R, 1656qRT-F/1656qRT-R; Table S1) were designed to specifically amplify part of each targeted gene Ccel_1229 and Ccel_1656, respectively. Real-time PCR was carried out on a CFX96 Real-Time System, C1000 Thermal cycler (BioRad). Thermal cycler was programmed for an initial step at 95°C for 5 s, 55°C for 10 s and 72°C for 1 s. Specificity of accumulated products was verified by using the melting-curve analysis. The Relative Expression Software Tool (REST) was used to calculate the relative expression of each gene in each condition using *rpoD*-like gene (locus Ccel_0541, amplified with primers RPO-F and RPO-R, table 1S), predicted to encode Sigma70 RNA-polymerase subunit. Quantification was performed in triplicate on each cDNA preparation.

### Purification of Cellulosomes, Separation of the Components by Gel Electrophoresis, and Western Blot Analysis

To collect the cellulosomes, *C. cellulolyticum* was grown in 800 mL of minimal medium containing cellulose or wheat-straw, until mid- to late- exponential phase. In the case of straw-based medium, the culture was incubated with cellulose (5 g/L; Sigmacell cellulose type 20) for 30 minutes prior to the purification step that was performed as previously described [11]. Cellulosomal components were separated by SDS-PAGE (10% [wt/vol] acrylamide) using 25 µg of purified cellulosomes as previously described [11].

For western blot analysis, the separated proteins were then electrotransferred to nitrocellulose membrane (Hybond™ ECL™ - GE Healthcare). After saturation, membranes were probed with polyclonal rabbit antibodies raised against the products of genes at loci Ccel_1230, Ccel_1234, Ccel_1237, and Ccel_1656. These antibodies were previously saturated with an extract of *E. coli* BL21(DE3) producing a CBM6-containing protein (encoded by gene at locus Ccel_1656) to increase specificity, in particular for antibodies raised against complete proteins. Antibodies were detected by using anti-rabbit horseradish peroxydase conjugate and a chemiluminescent substrate (GE Healthcare).

For liquid chromatography-tandem mass spectrometry (LC-MS/MS) analysis, gel lanes were cut into pieces of 15 mm^3^ (5 mm in width, 3 mm in length, and 1 mm in depth). The proteins of molecular mass between 160 and 45 kDa were then subjected to in-gel trypsin digestion and the peptides were eluted from the gel slices as previously described [43].

### Nano-LC-MS/MS Analyses

The nano-LC-MS/MS analysis of peptides derived from tryptic in-gel digestion was performed on a LTQ-Orbitrap mass spectrometer (Thermo Fisher Scientific, Waltham, MA) equipped with a nano-ACQUITY UPLC Waters, Milford, MA). Peptides were loaded onto a trapping column (nanoAcquity Symmetry UPLC column, C_18_, 5 µm, 180 µm by 20 mm; Waters) at a flow rate of 10 µl/min and washed for 3 min with 99% buffer A (0.1% acetic acid in distilled water). Peptides were then eluted and separated via an analytical column (nanoAcquity BEH130 UPLC column C_18_, 1.7 µm, 100 µm by 100 nm; Waters) with a decreased buffer gradient (from 99% buffer A to 60% buffer B (0.1% acetic acid, 99,9% acetonitrile) in a time frame of 80 min. The mass spectrometric analysis started with a full survey scan in the Orbitrap (*m*/*z* 300 to 2,000, resolution of 60,000) followed by a collision-induced dissociation and acquisition of MS/MS spectra of the four most abundant precursor ions in the LTQ. Precursors were dynamically excluded for 30 s, and unassigned charge states as well as singly charged ions were rejected.

The mass spectrometric data were subjected to database searching via Sorcerer and Sequest [44]. Charge state deconvolution and deisotoping were not performed. All MS/MS samples were analyzed using Sequest (Thermo Fisher Scientific, San Jose, CA, USA; version 27, revision 11). The searched database contained the target sequences, which include the complete proteome set of *C. cellulolyticum* H10 extracted from UniProtKB (http://www.uniprot.org/uniprot/?query=Clostridiumcellulolyticum&sort=score) and a set of common laboratory contaminants as well as a decoy data database. The precursor tolerance was set to 10 ppm, and tolerance for fragment ions was set to 1 atomic mass unit (amu). In these searches, we only allowed fully tryptic peptides, two missed cleavage sites, and methionine oxidation (+15.9949) as differential modification. Only b- and y-ion series were included in the database search. Data were merged in Scaffold (version 3.6.4). Filters in Scaffold were set as follows: XCorr scores of 1.9, 2.5, 3.8 and 3.5 for singly, doubly, triply and quadruply charged peptides, respectively and DeltaCn of 0.1. Only proteins identified with at least two peptides were included.

Quantification was done using spectral counts [45].

### Electrophoretic Mobility Shift Assay

To visualize binding of purified MBP-Δ116 to target DNA regions, electrophoretic mobility shift assays (EMSA) were performed by using the LightShift Chemiluminescent EMSA kit (Pierce). Fragments spanning 309 bp (R1) of the promoter region of the gene encoding the sensor (locus Ccel_1227); 347 bp (R2) of the promoter region of the gene encoding the regulator (locus Ccel_1228); 272 bp (R3), 429 bp (R4), and 345 bp (R5) of the promoter region of the first gene of *xyl-doc* gene cluster (locus Ccel_1229); and 246 bp (R6) and 240 bp (R7) of the promoter region of the gene at locus Ccel_1656 were amplified using the following primer pairs; 1227-1F/1227-1R, 1227rtD/1228rtR, 1229-1F/1229-1R, 1229-2F/1229-2R, 1229-3F/1229-3R, 1656-1F/1656-1R, and 1656-2F/1656-2R, respectively (Table S1).

The PCR products were purified from 1% agarose gel using MinElute gel extraction kit (Qiagen) and biotinylated at their 3′-ends as directed by the Biotin 3′ end DNA labeling kit (Pierce). For each probe, approximately 20 fmol of biotinylated DNA were incubated with various protein concentrations at 20°C for 20 min in a binding-reaction buffer including 10 mM Tris (pH 7.5), 150 mM KCl, 1 mM dithiothreitol, 30 ng/µL salmon sperm DNA, 0.1 µg/µL bovine serum albumin, 0.05% (v/v) Nonidet P-40 and 2.5% (v/v) glycerol. For competitive inhibition of the binding, unlabelled DNA was added to the binding reaction at a concentration 100 times higher than the biotin-labeled DNA. After the incubation, the DNA and protein mixtures were subjected to electrophoretic separation in a 5% non-denaturing precast-polyacrylamide gel (Bio-Rad) at 120 V for 1 hour and then transferred to a positively charged nylon membrane (Roche Applied Science) at 200 mA for 30 min. DNA was then cross-linked to the membrane at 180 mJ.cm^−2^ using a UV light cross-linker (UVP, HL-2000, Hybri-Linker). Finally, the biotin-labeled DNA was detected according to the LightShift Chemiluminescent EMSA kit protocol (Pierce).

## Supporting Information

Table S1 **Primers used in the study.**
(PDF)
