# Supplementary material for: A Two-Component System (XydS/R) Controls the Expression of Genes Encoding CBM6-Containing Proteins in Response to Straw in Clostridium cellulolyticum
Source: PLoS One. 2013 Feb 13;8(2):e56063. doi: 10.1371/journal.pone.0056063 (PMC3572039; doi:10.1371/journal.pone.0056063)
Supplement: Table S1 — Primers used in the study. (PDF) [file pone.0056063.s001.pdf]

TABLE S1. Primers used in the study.

Primer name	Sequence (5'- 3')[a]
1227-1F	GAGGGACACTTCTTACAGGCG
1227-1R	GGTGTATCATTAAAATCAAAGATGAGAGCT
1227rtD	CCAAGTTGGTTGGAAATGGT
1228DownNar	CATTAAGTGGCGCCCTATGCTTCACTCCTATACTCCTTTGG
1228Δ116UpBam	TAGGATCCAGAATTTAAAAGGAGGGATTAAAATGGAAAGGTA AAAGCTTTAATAATCG
1228rtD	GCCCAAGTCATTTTAGTACCATATT
1228rtR	AACTGATTTTGTATTCTCCGGAG
1229-1F	CCAAAGGAGTATAGGAGTGAAG
1229-1R	GCCAAAGAAAGTCAATACCTTATATAC
1229-2F	AATTCAAAAATTTATACCAATAC
1229-2R	GGCTAATTTGTCTTATAAAGGTAG
1229-3F	GGAGGTCGAAAAGTCTAGATAC
1229-3R	TGCAGACTATCCCATATTTTAC
1229qRT-F	GCCGTGATGTTGCCTTTATT
1229qRT-R	GTCCAATTCTGCCAACCACT
1229rtD	TTGGAAACGGTGCTGGAGG
1229rtR	TTGCAGACTATCCCATATTTTACC
1230GH10-NdeF	TTTGTTCATATGATGGCCACAGGAAAG
1230GH10-XhoR	TTTGGCTCGAGTGAAAATGCACTTCTTGTTT
1230rtD	GGGGTTCACAATATCCAAACTGG
1230rtR	GGCTGCACAGAACTTCCCG
1231rtD	GCCACTGTCATACTCTTCGTTC
1231rtR	GGCGACATTGACCCGACAG
1232rtD	GAGTCCAGAGTAACATTCCGC
1232rtR	CTGCATGGTATCGGTGGAAAT
1233rtD	TTATGCAGATATTAACTGGTTCCG
1233rtR	CAAGCATTGCCTACCGTAAC
1234-NcoF	CAGCCATGGCGGCAAACCCAAATCCGTCATG
1234rtD	GGAGATTACAGCGTATACAAGAGCAT
1234rtR	ATTTCTGATAAGGCCCAAATCCC
1234-XhoR	CCCCCCTCGAGCGCCTGAGCAGGGAATTTTG
1235rtD	ATGCTATTCAACAACAGATATGG
1235rtR	AGATTTTGGCTCGGGAGCAT
1236rtD	GTCTTTTAGTGCAGATAATTCG
1236rtR	AGCTATTATGGAGGGTGGGAA
1237-NdeF	TCTTCTACATATGGATAACGGTCTTGCAAAAACA
1237rtD	GACCAGCGGAGGCAATATTGAGATTA
1237rtR	TTCCAGGCAAGAGTAGCA
1237-XhoR	CCCCCCTCGAGTGCTGCCCCCTGCCC
1238rtD	CCAACGGAGAAATTAACGCA
1238rtR	GGAATAGTGAAGCACAGGCCAC
1239rtD	CGGTGTAATTGTGGGAGTTG
1239rtR	TCCCCACAGCTGACAATACA
1240rtD	GGTCACAATACACTGGTAAATCAACTA
1240rtR	GGCCGGGAAAGGTAAAAACACT

1241rtD	CTGTGCCCGTGGAAATTTC
1241rtR	TGGTCAACCGGGCATG
1242rtD	AGGTGCCAAGGTAATAGCCCC
1242rtR	GGCACTGCAAATCAGACACGTCT
1243rtR	TGGTTACAGCTACATATCTTTGATAAA
1656-1F	GCTGCGGAATAAGGTATTAAC
1656-2F	GAGGCTAAAATCTAAGAGAATAAG
1656-1R	GCAATTAGAAACTGATTAAGAAGG
1656-2R	CTTAAAAGAGGAGGACAAGTATG
1656qRT-F	TTGTCCTCCTCTTTTAAGTTATATT
1656qRT-R	ACTGGTTAATCGATAGTCTCCTCCTCTTAAAT
1656rtD	TTGTCCTCCTCTTTTAAGTTATATTA
1656rtR	TCACTGCTGCTTACGTTT
1656UNK-NdeF	GGAATTCCATATGACGCAGGCAGCCTATTACG
1656UNK-XhoR	CCGCCGCTCGAGAGCGGAACCGCTCACTCTCAG
catdir	ACCATCGATGGTCCTTGTTCTCCTTTTCTCTG
catrev	AACGAGCTCGGGAGGCCTAGAGAAAAGGAGAACAAGAA
EBS1d-xydR	CAGATTGTACAAATGTGGTGATAACAGATAAGTCGCGGTCAGTAACTTACCTTTCTTTGT
EBS2-xydR	TGAACGCAAGTTTCTAATTTCGATTTTTATTCGATAGAGGAAAGTGTCT
EBS Universal	CGAAATTAGAAACTTGCGTTCAGTAAAC
IBS-xydR	AAAAAAGCTTATAATTATCCTTAATAAACGCGGTCGTGCGCCCAGATAGGGTG
MBP-Δ116F	ATATGGATCCGAAAAGGTAAAAGCTTTAATAATCGAT
MBP-Δ116R	TAAACTGCAGCTATGCTTCACTCCTATACTCCTTTG
RPO-F	AAACATAGTCAAGAAAGTAGAAAAG
RPO-R	CTATACTAACAACCAGCCTTAAG
tetd	TCCCCCGGGATTTGGTACTTGAAAAG
tetr	CATATCGATAGAGCCGATAAAATGAGATTAATAC

[a] Relevant restriction sites are underlined
